# Anti-inflammatory effects of green soybean extract irradiated with visible light

**DOI:** 10.1038/srep04732

**Published:** 2014-04-22

**Authors:** Keiko Tanaka, Yasushi Ohgo, Yuki Katayanagi, Kensuke Yasui, Shigeru Hiramoto, Hiroyuki Ikemoto, Yumi Nakata, Noriyuki Miyoshi, Mamoru Isemura, Norio Ohashi, Shinjiro Imai

**Affiliations:** 1 Health Care Research Center, Nisshin Pharma Inc., 5-3-1, Fujimino, Saitama, 356-8511 Japan; 2 Graduate School of Nutritional and Environmental Sciences, University of Shizuoka, 52-1 Yada, Suruga-ku, Shizuoka, 422-8526 Japan; 3 These authors contributed equally to this work.

## Abstract

We conducted a preliminary investigation of the effects of visible light irradiation on plant extracts, and we observed a strong suppressive effect on interleukin (IL) 2 expression with the inhibition of c-Jun amino-terminal kinase (JNK) phosphorylation in Jurkat cells by visible light irradiation to ethanol extract from green soybeans (LIEGS). This effect was produced only by extracts from green soybeans (*Glycine max*) and not other-color soybeans. LIEGS suppressed the lipopolysaccharide-induced IL-6, IL-12 and TNF-α expression levels in human monocyte THP-1 cells in a concentration-dependent manner. LIEGS was applied for 8 weeks to NC/Nga mice. LIEGS suppressed the development of atopic dermatitis (AD)-like skin lesions and reduced the dermatitis scores of the mice. The light irradiation changed the various types of small-molecule compounds in extracts. Visible light irradiation to daidzein with chlorophyll b induced a novel oxidative product of daidzein. This product suppressed IL-2 expression in Jurkat cells.

The light irradiation of food can oxidize several compounds in the food, often resulting in many negative effects on human health. Other compounds, such as vitamin D, are activated by ultraviolet irradiation^1^. Irradiation at visible blue and UV-A wavelengths of green tea leaves caused a larger increase in antioxidant activity than other treatments^2^. We suspected that the light irradiation of some plant extracts might have positive effects on human health. We performed a preliminary investigation of the effects of visible light irradiation on some edible plant extracts. As an index of the effect, we used IL-2 suppression in Jurkat cells (a human T-cell-like cell line), and we confirmed a strong suppressive effect of visible light irradiation to green soybean ethanol extract on IL-2 expression in Jurkat cells.

Soybean research has indicated that there are various elements in foods made with soybeans that moderate or lessen the development potential for chronic diseases^3^. Examples include the hypocholesterolemic effects of soy phytoestrogen^4^,^5^,^6^, the cancer risk-reducing properties of the isoflavonoids^7^,^8^ and the saponins^9^, and the xenobiotic effect of genistein^10^. It appears that there are health benefits to including soybeans in one's diet. Soybeans are commercialized in a wide variety of colors and tones — from yellow to black through green, brown and red. The yellow pigmented soybean is the most abundant and harvested soybean.

The health benefits of soybeans have been reported to differ according to differences in color. For example, the extracts from black soybeans have a longer low-density lipoprotein oxidation lag time compared to that from yellow soybeans because of the high total polyphenol content in its seed coat^11^. The brown soybean seed coat was shown to have high radical-scavenging activity due to a highly polymerized proanthocyanidin component^12^. Another study showed that a diet enriched in soybean isoflavones and green tea enhanced the immune function of ovariectomized mice (natural killer activity and lymphoproliferation) compared to control mice^13^.

We recently clarified that water extracts from green soybeans have inhibitory activity for pollinosis with regard to the production of IgE, as well as the ability to regulate B-cell apoptosis, probably by suppressing the expressions of BAFF and APRIL^14^.

A soybean seed is usually green before it is harvested and becomes yellow during maturation. In the present study, the terms “yellow” and “green” soybean refer to the seed coat cotyledon color of each mature soybean (*Glycine max*) at post-harvest. We evaluated and compared the effects of green and yellow soybean extracts with or without visible light irradiation on anti-inflammatory activity.

## Results

### Visible light irradiation to ethanol extract from green soybean suppressed the IL-2 expression levels in Jurkat cells

**Figure 1:**
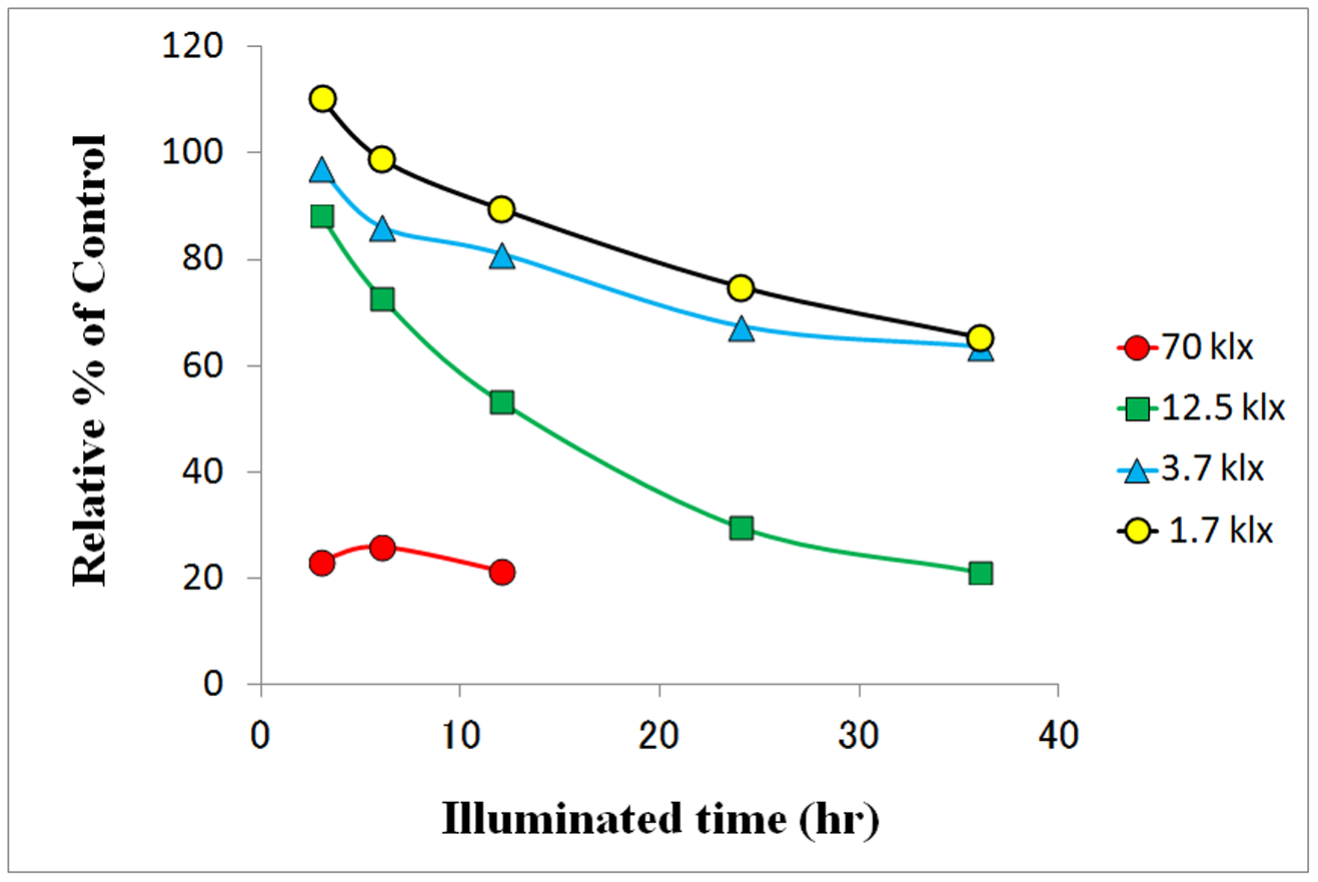
LIEGS suppressed the calcium ionophore and phorbol ester-induced IL-2 expression levels in Jurkat cells. IL-2 levels were measured by ELISA in the supernatant of Jurkat cell culture (1 × 10^5^/mL) with 50 μg/mL of the soybean extracts that were with irradiated visible light at a specified distance between the fluorescent lamp and the extract at different times. The IL-2 levels are expressed as the mean concentrations.

We measured the calcium ionophore and phorbol ester-induced IL-2 levels by ELISA in the supernatant of Jurkat cell culture with 50 μg/mL of the soybean extracts that were irradiated with visible light at varying distances from a fluorescent lamp to the extract at different times. When green soybean extracts were irradiated longer and at the shortest distance, the IL-2 levels were more suppressed (Fig. 1).

### Difference of cultivate variety of soybean seeds

**Figure 2:**
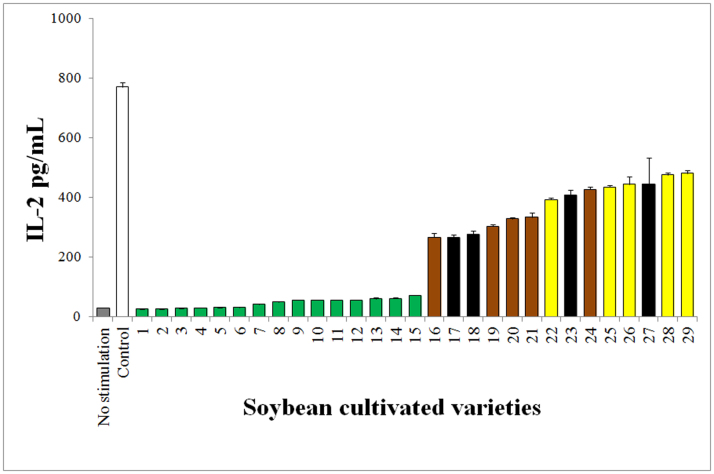
Differences in soybean seed color. Here, 29 cultivated varieties of mature soybean seeds were extracted with EtOH, and then the extracts were irradiated by visible light at 70 klx for 48 h. Jurkat cells were incubated with the light-irradiated extracts for 18 h, and then the calcium ionophore and phorbol ester-induced IL-2 levels of the culture supernatants were measured by ELISA. The IL-2 levels are expressed as the mean ± SE concentrations. The soybean cultivars were classified by cotyledon colors, which were green (green bars), yellow (yellow bars), black (black bars) and other color (brown bars).

Twenty-nine cultivated varieties of mature soybean seeds were extracted with ethanol, and then the extracts were irradiated by visible light at 70 kilolux (klx) for 48 h. Jurkat cells were incubated with the light-irradiated extracts for 18 h, and then the calcium ionophore and phorbol ester-induced IL-2 levels of the culture supernatants were measured by ELISA. Only the varieties with green color cotyledons suppressed IL-2 expression in the culture supernatant (Fig. 2).

### LIEGS suppressed IL-2 expression by blocking JNK signaling

**Figure 3:**
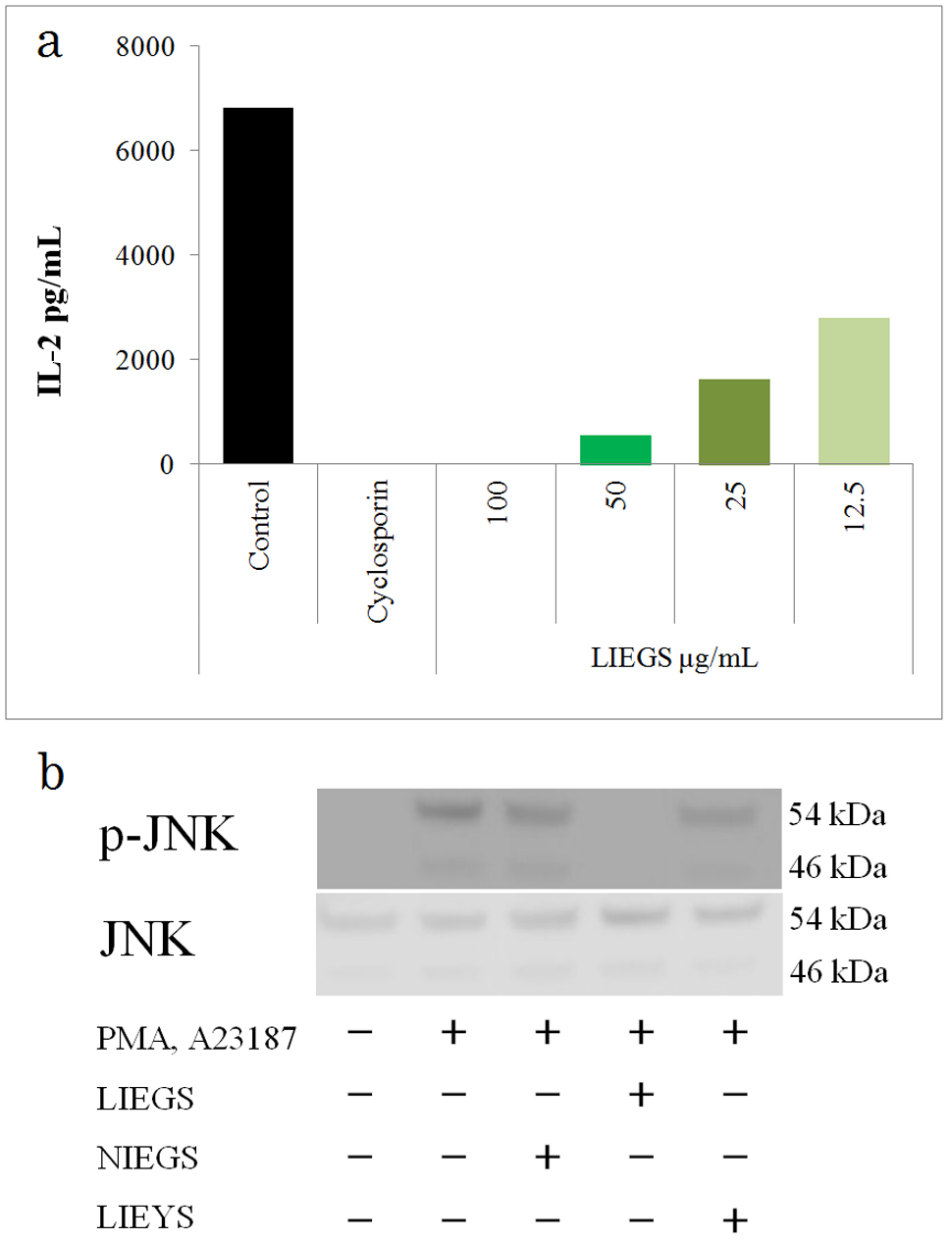
LIEGS suppressed the IL-2 expression by blocking JNK signaling. LIEGS suppressed the calcium ionophore and phorbol ester-induced IL-2 expression levels in Jurkat cells in a concentration-dependent manner (a). Differences compared with the control were analyzed by Student's t-test. *p < 0.05, **p < 0.01. Phosphorylation of JNK was analyzed by Western blotting. Stimulated Jurkat cells were lysed and total protein concentrations were determined from cellular extracts. Equal amounts of total proteins (60–80 μg) were separated by 10% SDS-PAGE and transferred onto PVDF membranes for the detection of each IL-2 signal transduction proteins using phosphorylated protein-specific antibodies. After 30-min stimulation by calcium ionophore and phorbol ester, the phosphorylation of JNK was significantly increased, and this phosphorylation was blocked by the simultaneous treatment with LIEGS. Representative Western blots are shown to (b). Full-length blots are presented in Supplementary Figure 1.

The LIEGS suppressed the calcium ionophore and phorbol ester-induced IL-2 expression levels in the Jurkat cells in a concentration-dependent manner (Fig. 3a). Non-irradiated ethanol extracts from green soybean (NIEGS) and visible light irradiated ethanol extracts from yellow soybean (LIEYS) barely suppressed the gene expression levels of the cytokines. After the 30-min activation by calcium ionophore and phorbol ester, the phosphorylation of JNK was significantly increased, and this phosphorylation was blocked by the simultaneous treatment with LIEGS (Fig. 3b) but not NIEGS. ERK1, p38 MAPK and IKK phosphorylation levels were not affected by LIEGS (data not shown).

### Anti-inflammatory activity of LIEGS in culture cells

**Figure 4:**
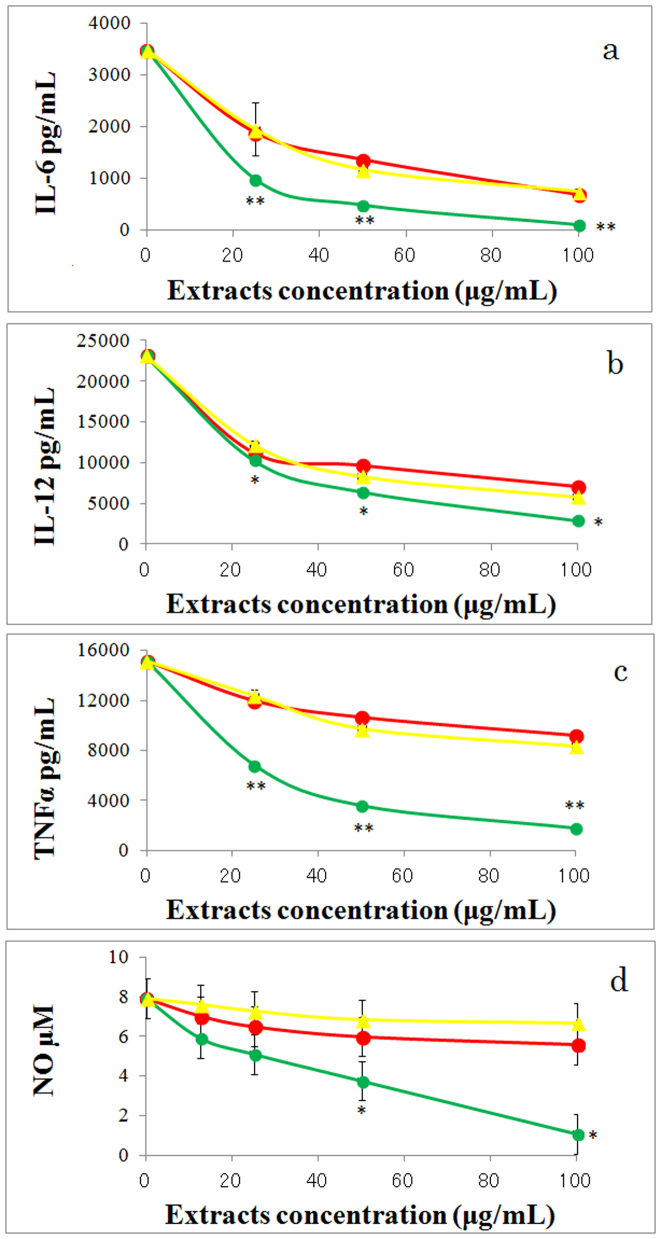
LIEGS suppressed the pro-inflammatory cytokine. LIEGS suppressed the expression levels of the LPS-induced pro-inflammatory cytokines IL-6 (a), IL-12 (b) and TNFα (c) in THP-1 cells in a concentration-dependent manner. Line colors: LIEGS (green), NIEGS (red) and LIEYS (yellow). The cytokine levels are expressed as the mean ± SE concentrations. LIEGS suppressed the LPS-induced NO levels in RAW264.7 cells in a concentration-dependent manner (d). Differences compared with the control were analyzed by Student's t-test. *p < 0.05, **p < 0.01.

LIEGS suppressed the LPS-induced IL-6, IL-12, and TNFα expression levels in human monocyte THP-1 cells in a concentration-dependent manner (Fig. 4a–c), whereas NIEGS and LIEYS produced little suppression of the gene expression levels of the cytokines. LIEGS suppressed the LPS-induced nitric oxide (NO) levels in mouse macrophage RAW264.7 cells in a concentration-dependent manner (Fig. 4d), whereas NIEGS and LIEYS produced little suppression of the NO levels of the cytokines.

### Effect of LIEGS on the atopic dermatitis mouse model

**Figure 5:**
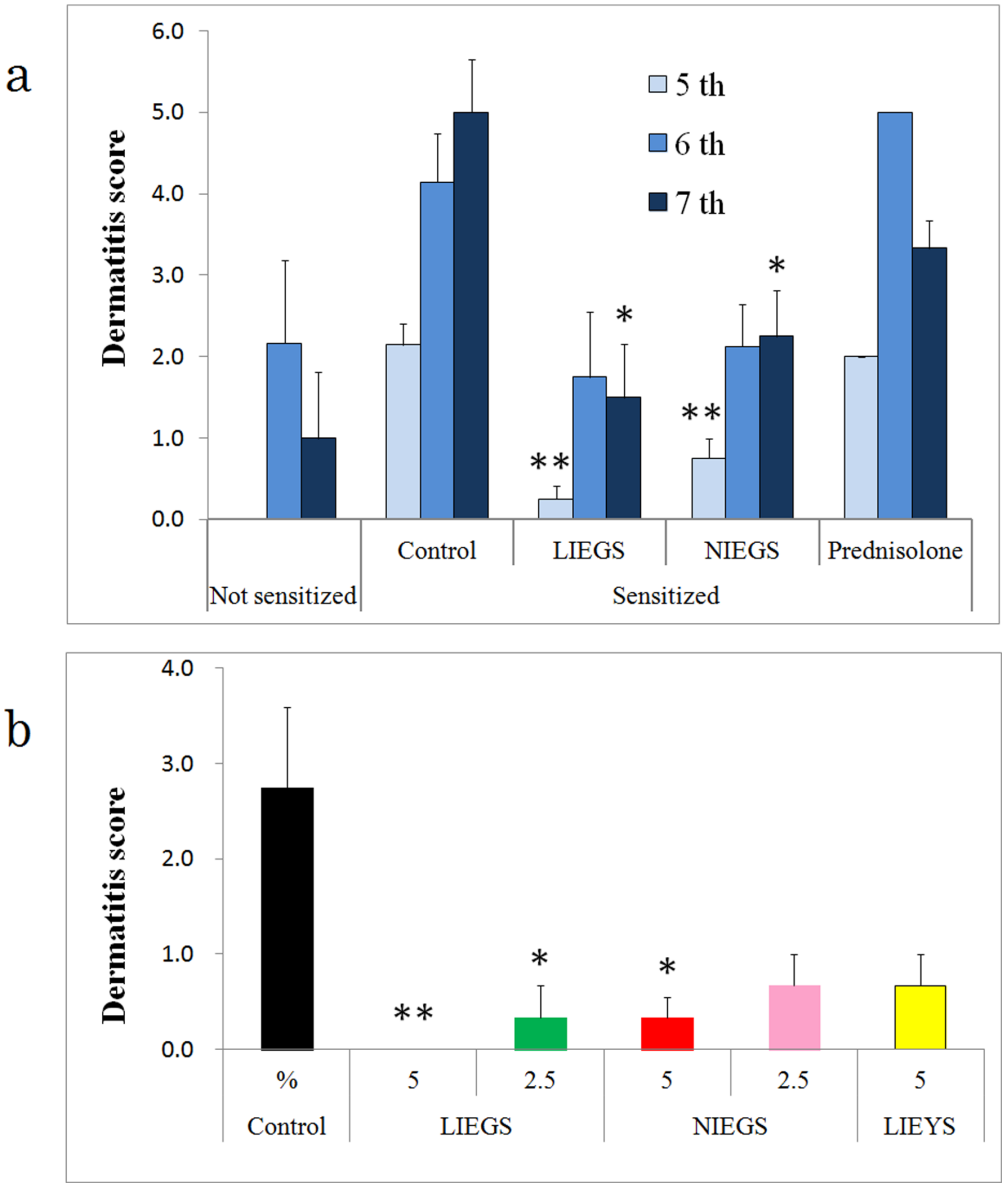
Effects of LIEGS and NIEGS on atopic dermatitis (AD) in AD-sensitized NC/Nga mice. (a) The skin severity score was evaluated after the 5th, 6th and 7th sensitization of antigens. (b) The dose dependency of LIEGS and NIEGS was evaluated at the 5th sensitization of antigens. Values are expressed as mean ± SE. Differences between the groups were analyzed by Steel's multiple comparison test. *p < 0.05, **p < 0.01.

Skin inflammation with typical itching behavior was observed in the control NC/Nga mice at 8–9 weeks of age. The oral intake of LIEGS and NIEGS significantly suppressed the increase in clinical skin severity score at the fifth sensitization of antigen (Fig. 5a). However, the prednisolone intake group did not show a suppression of the increase in clinical skin severity score. In the second animal experiment, the LIEGS and NIEGS intake groups showed suppressed increases in the clinical skin severity score in a dose-dependent manner (Fig. 5b); the 5% LIEGS intake group showed significant suppression. In the LIEYS intake group, the increase in clinical skin severity score was also suppressed but not significantly.

### Analysis of different components in LIEGS and NIEGS

**Figure 6:**
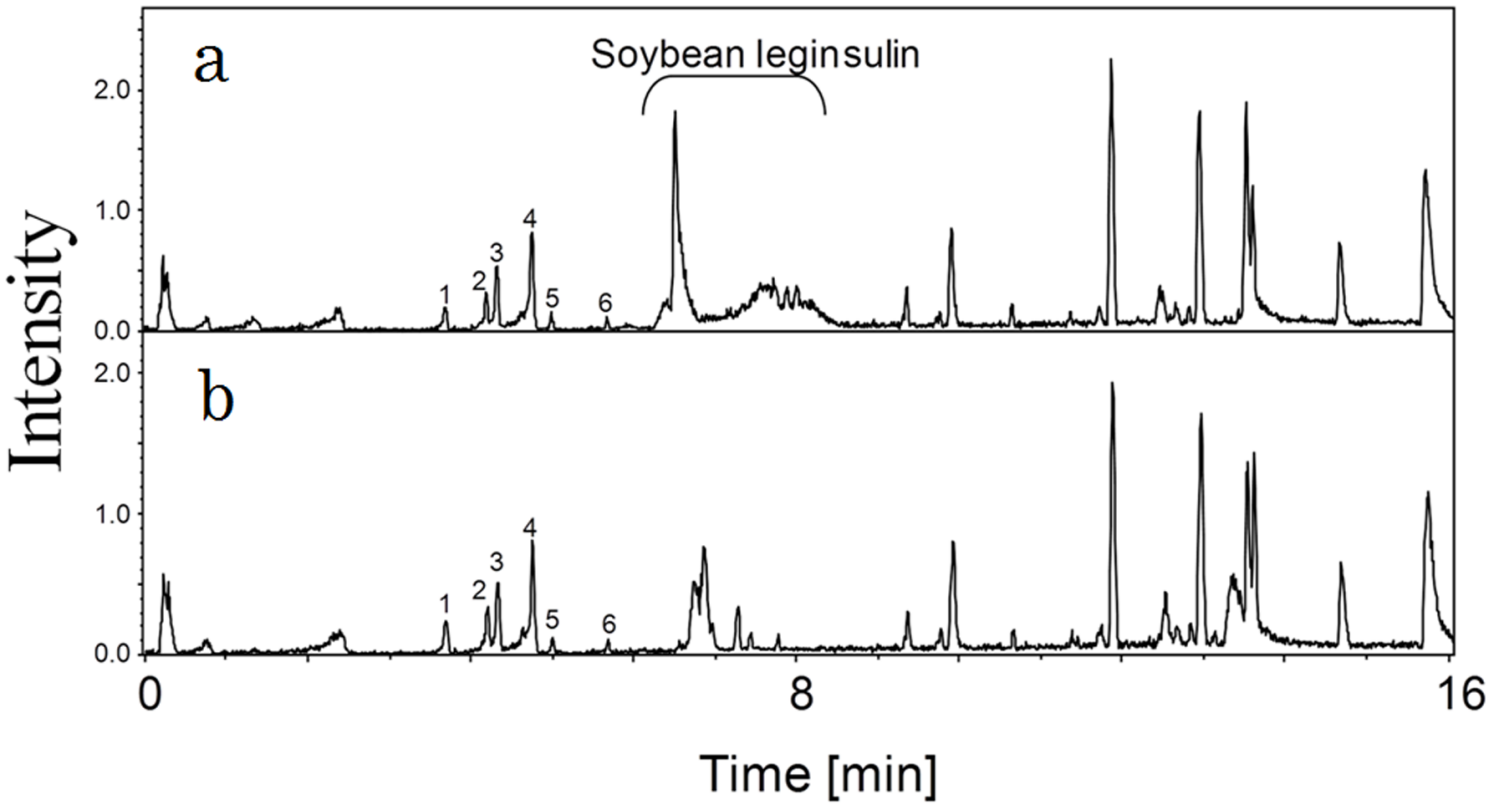
Analysis of different components in LIEGS and NIEGS. Base ion peak chromatograms obtained by UPLC-TOF-MS analyses of LIEGS (n = 3, (a)) and NIEGS (n = 3, (b)). Daidzin (1), genistin (2), malonyldaidzin (3), malonylgenistin (4), daidzein (5) and genistein (6) were eluted as indicated by peak numbers.

Our analyses of LIEGS revealed 552 MS ion peaks. The base ion peak chromatograms indicated that MS ions detected at 6.5–8.5 min were clearly varied by the visible light irradiated sample (Fig. 6). We identified these peaks derived from soybean leginsulin and its modified forms. Isoflavones including daidzein, genistein and their glycosides and malonylated forms were eluted at 3.6–5.8 min, but the levels of these compounds were not significantly altered by the visible light irradiation. Among other unidentified peaks, the levels of two MS ion peaks were significantly higher in the visible light irradiated group: m/z 814.55 at 13.12 min, and 941.50 (471.25, z = 2) at 7.76 min.

### Chemical and biochemical characterization of the relevant compounds

**Figure 7:**
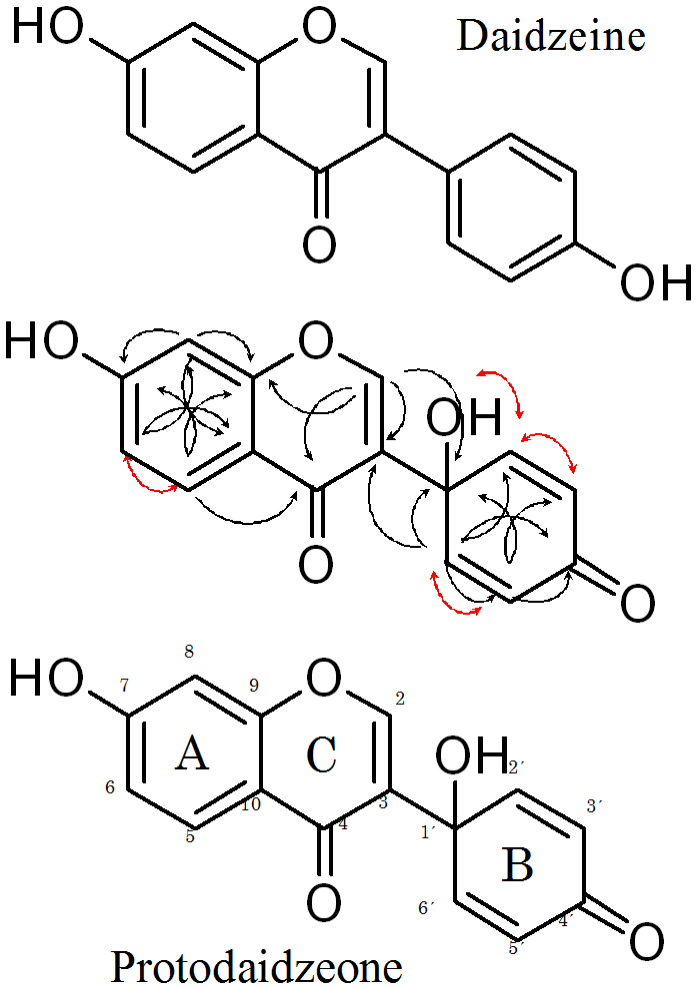
Structures of active compounds. The chemical structures of daidzein and protodaidzeone. Key HMBC (black arrow) and^1^H-^1^H COSY (red arrow) correlation of protodaidzeone.

Visible light irradiation to daidzein with chlorophyll b induced NO suppression in RAW264.7 cells. The active compound was isolated as a white amorphous solid from the reaction mixture using preparative HPLC. EI-MS of this compound gave an [M + H]+ peak at 271.0606, which was consistent with the molecular formula of C_15_H_10_O_5_, showing it was an oxygen adduct of daidzein. The comparison of^1^H-NMR and^13^CNMR data between this compound and daidzein showed that this compound has very close chemical shifts to those derived from the A and C rings in daidzein, but this compound does not have signals similar to those from the B ring in daidzein: one of the aromatic methine-signals in daidzein disappeared in this compound, an oxygen-bonding aliphatic quaternary carbon signal at δC 66.37 newly appeared in this compound, and other aromatic carbon-derived ^13^C-NMR signals from the C ring in daidzein were shifted to a lower field in this compound. These data suggested that the B ring in daidzein was modified to a different structure in this compound (Table 1). The structure of this compound was determined as 7-hydroxy-3-(1-hydroxy-4-oxo-2,5-cyclohexadienyl) chromen-4-one by the detailed analysis of correlation signals in HMQC, HMBC and ^1^H-^1^H-COSY as shown in Figure 7.

The structural difference between this compound and daidzein was seen in the C ring: the C ring in daidzein was modified to a 1-hydroxy-4-oxocyclohexa-2,5-dienyl group in this compound via a chlorophyll-catalyzed oxidative reaction of daidzein. This compound was a novel compound, and therefore it was named protodaidzeone according to the naming of protoapigenone, a compound of the same class as this compound, which had been reported as antitumor^15^.

Protodaidzeone was present in a very small amount in LIEGS according to our LC-MS analysis (data not shown). Protodaidzeone suppressed the LPS-induced NO levels in the RAW264.7 cells and the calcium ionophore and phorbol ester-induced IL-2 expression levels in the Jurkat cells in a concentration-dependent manner. The respective IC_50_ values of protodaidzeone were 842 nM and 294 nM.

## Discussion

We report here that visible light-irradiated green soybean extracts acted in an anti-inflammatory manner and simultaneously improved atopic dermatitis in an animal model. Genistein, the principal isoflavone in soy, has been identified as a protein tyrosine kinase (PTK) inhibitor^16^ that possesses anti-inflammatory properties in vitro^17^,^18^,^19^. In another study, the pretreatment of RAW 264.7 cells with genistein inhibited the release of both LPS-stimulated TNF-α and IL-6^20^. The injection of a high dose of genistein (50 mg/kg) to mice attenuated LPS-induced acute lung responses through the inhibition of NF-κB activation, reducing lung injury and lethal toxicity^21^. The oral intake of genistein by rats had anti-inflammatory effects *in vivo*^22^.

These studies suggested that the compounds with anti-inflammatory activity in green soybean extracts may be isoflavones. However, genistein did not suppress IL-2 expression in Jurkat cells^23^, and in the present study, the levels of isoflavones were not significantly altered by the visible light irradiation. LIEGS suppressed IL-2 expression by blocking JNK signaling. But soy isoflavones do not affect JNK signaling in inflammation. The levels of soybean leginsulin and its modified forms were altered by the visible light irradiation. It is not clear whether leginsulin has anti-inflammatory activity. We suspect that leginsulin is not absorbed by the intestine in its maintained active form.

We attempted to identify the active compounds in LIEGS. However, the active fractions were dispersed, and we were not able to confirm the active compounds. Visible light irradiation to the ethanol extracts of green soybean may induce modifications of many types of compounds. Visible light irradiation to the ethanol extracts of yellow soybeans with chlorophyll b induced NO suppression in RAW264.7 cells (data not shown). Therefore, we confirmed the changes of specific compounds in green soybeans by visible light irradiation. Visible light irradiation to daidzein with chlorophyll b induced NO suppression in RAW264.7 cells. An active compound in this irradiated solution was identified as protodaidzeone, an oxidative product of daidzein. This product was present in a very small amount in LIEGS according to our LC-MS analysis. This product may share an active part of LIEGS. The visible light irradiation with chlorophyll to soybean extracts may induce oxidization in many types of compounds. Some of these oxidized compounds may suppress inflammation.

We recently clarified that water extracts from green soybeans have inhibitory activity for pollinosis^14^ with regard to the production of IgE, but the active compounds were not identified. In the present study, the total IgE levels in NC/Nga mice were measured by ELISA (data not shown). The total IgE levels in all of the groups were increased by sensitization with Biostir AD, with little difference among the groups. However, LIEGS strongly suppressed the dermatitis, indicating that LIEGS suppresses only inflammation and not type I allergic responses, which depend on IgE.

Visible light irradiation to ethanol extracts of green soybean had a marked effect on the anti-inflammatory activities. This effect was produced only by the extract of soybeans with green cotyledons. LIEGS showed suppressive activity with regard to the development of AD-like skin lesions and reduced the dermatitis scores of the back skin of mice. Our data indicate the great potential of LIEGS as a suppressor of activated T cells and macrophages, ultimately as an anti-inflammatory reagent.

## Methods

### Materials

Soybean samples were purchased from local markets in Shizuoka, Japan. Briefly, ethanol extracts were obtained by the maceration of dried soybean powder (10 g) in a flask with 100 mL ethanol, followed by stirring for 2 h at 25°C and then filtration on a glass filter. Ethanol extracts were concentrated in a vacuum. The dried-up extractions were dissolved in dimethyl sulfoxide (DMSO; Wako Pure Chemicals, Tokyo) or ethanol (for *in vivo* studies).

### Light irradiation

Ethanol extracts of soybeans were continuously irradiated at a distance from a fluorescent lamp (FML36EXN, Panasonic, Osaka, Japan). Each illuminance (klx) was measured by a T-10A Series illuminance meter (Konica Minolta, Tokyo).

### Cell culture treatments

Human Jurkat, THP-1 and RAW264.7 cells were routinely cultured in RPMI-1640 medium supplemented with 10% fetal calf serum (FCS), 2 mM l-glutamine, 50 μg/mL penicillin-streptomycin at 37°C in a humidified chamber containing 95% air and 5% CO_2_. Cell viability was assessed by a trypan blue exclusion test. Cell numbers were determined with a hemocytometer. IL-2 was induced by the addition of A23187 (Calcium ionophore; Merck, Darmstadt, Germany; final concentration 500 pM) and TPA (Merck, 12-o-tetradecanoylphorbol-13-acetate; final concentration 500 ng/mL) in the culture medium of Jurkat cells. IL-6, IL-12, and tumor necrosis factor-alpha (TNF-α) were induced by addition of 100 ng/mL (final concentration) of lipopolysaccharide (from *E. coli*, Sigma-Aldrich, St. Louis, MO) in the culture medium of THP-1 cells.

### Cytokine measurement

To determine the concentrations of cytokines in the supernatant of Jurkat cells and THP-1 cells, a sandwich enzyme-linked immunosorbent assay (ELISA) was performed using DuoSet (R&D SYSTEMS, Minneapolis, MN) human IL-2, IL-6, IL-12, and TNF-α according to the manufacturer's instructions. All assays were performed in triplicate. The concentration of each protein was calculated from the standard curve.

### Nitric oxide inhibition assay

NO production was assayed indirectly by measuring the accumulation of nitrite plus nitrate according to a spectrophotometric method^24^. Briefly, light irradiation to ethanol extract from green soybean (LIEGS) and visible light irradiated daidzein with chlorophyll b were dissolved in DMSO, and were further diluted with the DMEM to give a final DMSO concentration of 0.25%, which had no effect on the growth of RAW 264.7 cell in the assay. RAW 264.7 cells (1.2 × 10^6^) were seeded in each well of a 24-well plate. The cells were treated with 500 ng/mL of lipopolysaccharide (LPS) and various concentrations of assayed compound, and the plates were incubated for an additional 18 h at 37°C. To measure the nitrite plus nitrate concentration in medium, we collected aliquots of 100 μL of the culture supernatant, mixed them with standard Griess reagent, and incubated them at 37°C for 15 min to form a purple azodye. The absorption at 540 nm was determined using a microplate reader.

### Western blotting

Stimulated Jurkat cells were lysed and the total protein concentrations were determined from cellular extracts. Equal amounts of total proteins (60–80 μg) were applied to 10% SDS-PAGE and transferred onto PVDF membranes for the detection of each IL-2 signal transduction proteins ERK1, p38 MAPK, IKK and JNK using the phosphorylated protein-specific antibody (Cell Signaling Technology Japan, Tokyo) each.

### Animal experiments

All experiments were approved by our institutional Animal Care and Use Committee and were performed in accord with institutional guidelines. Six-week-old male NC/Nga mice were purchased from Japan SLC (Hamamatsu, Japan) and housed in an air-conditioned room maintained at 24° ± 2°C and 55° ± 15% humidity.

In the first animal experiment, mice were randomly divided into five groups: (1) normal controls that did not receive antigen sensitization, (2) the antigen-sensitized control group, (3) antigen-sensitized mice whose feed contained 5% of visible light irradiation to ethanol extract from green soybean (LIEGS), (4) antigen-sensitized mice whose feed contained 5% of non-irradiated ethanol extracts from green soybean (NIEGS), and (5) antigen-sensitized mice whose feed contained 0.0002% of prednisolone (Takeda Pharmaceuticals, Osaka, Japan) as a positive control, and then fed the AIN76^25^-based experimental soybean extracts or control diets starting on Day 0.

In the second experiment, mice were randomly divided into six groups: (1) the antigen-sensitized control group; antigen-sensitized mice fed 5% (2) and 2.5% (3) of LIEGS; 5% (4) and 2.5% (5) of NIEGS; and 5% of visible light irradiated ethanol extracts from yellow soybean (LIEYS) (6).

### Sensitization

Atopic dermatitis (AD)-like skin lesions were induced in 8-week-old male NC/Nga mice using *Dermatophagoides farinae* (*D. farina*e) extract as described by the manufacturer^26^. An ointment of *D. farinae* extract (Biostir AD) was purchased from Biostir Inc. (Hyogo, Japan) and applied once per week for 7 weeks. Briefly, the hair on the upper back was shaved, and then 200 μL of 10% (w/v) sodium dodecyl sulfate (SDS) was applied for barrier disruption to the shaved dorsal skin. After 1 h, 50 mg of Biostir AD was applied to the skin.

### Dermatitis score

The severity of dermatitis was investigated macroscopically. The back skins were scored according to the following symptoms: rubefaction, erosion, and armpit ulcers. The severity score was defined as the sum of individual scores, graded as follows: 0 (no symptom), 1 (rubefaction in up to four places), 2 (rubefaction in five or more places), 3 (slight erosion), 4 (extreme erosion), 5 (extreme erosion with one site of armpit ulcers), and 6 (extreme erosion with two or more sites of armpit ulcers).

### UPLC-TOF-MS analyses

Ten microliters of freeze-dried samples dissolved in 70% EtOH (1 mg/mL) were injected into an ultra-performance liquid chromatography–time of flight mass spectrometry (UPLC-TOF-MS) system, which consisted of an Acquity™ UPLC (Waters, Milford, MA) and a MicroQTOF II mass spectrometer (Bremen, Germany). UPLC separation was performed with an Acquity UPLC™ BEH C18 column (1.7 μm, 50 mm × 2.1 mm i.d., Waters) at 40°C, using solvent A (0.1% formic acid in water) and solvent B (MeCN containing 0.1% formic acid).

Samples were eluted from the column using a linear gradient of 5% solvent B from 0.2 min to 90% solvent B at 13.16 min. The flow rate of the mobile phase was 0.4 mL/min. The TOF-MS was operated in the positive ion mode using an electrospray ionization source (ESI+). The detector conditions were as follows: capillary voltage at 4500 V, nebulizer at 1.8 bar, drying gas flow at 10 L/min, drying gas temperature 200°C, and the mass range between 50 and 2500 m/z. All analyses were performed using a low-concentration tuning mix (Agilent Technologies, Palo Alto, CA) to calibrate accurate mass. MS peak data from UPLC-TOF-MS analyses were subjected to Signpost™ software (Reifycs, Tokyo) for peak detection and integration.

### Isoflavone conversion and purification

Five mL of Daidzein in DMSO (12 mg/mL) with 0.2 mg/mL of chlorophyll b was continuously irradiated for 6 days at 2 klx/h. The irradiated solution was applied for reverse phase (RP) HPLC. The RP-HPLC analysis was performed on an Inertsil C8 column (GL Sciences, Tokyo; 250 mm × 20 mm i.d. × 5 μm). The sample was separated by RP-HPLC using 0.1% acetic acid and 10% acetonitrile (v/v) in water as the eluent (A), and 0.1% acetic acid and 35% acetonitrile as the eluent (B). The initial mobile phase composition was 100% A, followed by a linear gradient to 100% B in 50 min. The flow rate was 0.7 mL/min, the column temperature was set at 40°C, and the sample injection volume was 500 μL. The UV detection was performed at 190 nm. The active fraction at 26-min retention time was collected and dried *in vacuo* for analysis.^1^H and ^13^C-NMR spectra were measured on a Biospin AVANCE III 400 instrument (Bruker, Bremen, Germany)), operating at 400 (^1^H) and 100 (^13^C) MHz. Dimethyl sulfoxide-d5 was used as the solvent with TMS as the internal reference.

### Statistical analysis

The data are presented as the mean ± standard error (SE). Statistical analyses were performed using Steel's multiple comparison test or Student's t-test. Differences were considered significant when P-values were less than 0.05.

## Author Contributions

S.I., M.I. and N.O. conceived the experiments. K.T. and Y.K. performed the *in vivo* experiments and analyzed the data together with S.I. Y.O., K.T., Y.K., K.Y., Y.N. and R.F. performed the *in vitro* experiments and analyzed the data together with S.I. H.I. prepared material. S.H. analyzed chemical structure of compound. N.M. performed UPLC-TOF-MS analyses. S.I. wrote the paper. All authors discussed the results and commented on the manuscript.

## Supplementary Material

Supplementary Information Supplementary figure 1

## Figures and Tables

**Table 1:** ^1^H and ^13^C-NMR data of daidzein and protodaidzeone

		*Daidzein		Protodaidzeone	
Part (ring)	Position	δC	δH	δC	δH
C	2	152.78	8.29	154.61	8.36
C	3	122.52		121.89	
C	4	174.66		173.44	
A	5	127.26	7.97	126.57	7.79
A	6	114.90	6.94	115.52	6.89
A	7	162.46		163.20	
A	8	102.06	6.86	102.04	6.83
A, C	9	157.40		157.52	
A, C	10	116.61		116.01	
B	1′	123.46		66.37	
B	2′	130.04	7.39	149.67	6.77
B	3′	115.01	6.81	127.29	6.13
B	4′	157.12		185.54	
B	5′	115.01	6.81	127.29	6.13
B	6′	130.04	7.39	149.67	6.77
A	7-OH		10.76		
B	1′-OH				6.50
B	4′-OH		9.52		

*The NMR data of daidzein in pyridine-d5 are from the actual measurements.
