# Supplementary material for: Anti-inflammatory effects of green soybean extract irradiated with visible light
Source: Sci Rep. 2014 Apr 22;4:4732. doi: 10.1038/srep04732 (PMC3994445; doi:10.1038/srep04732)
Supplement: Supplementary Information — Supplementary figure 1 [file srep04732-s1.doc]

Anti-inflammatory effects of green soybean extract irradiated with visible light

Keiko Tanaka, Yasushi Ohgo, Yuki Katayanagi, Kensuke Yasui, Shigeru Hiramoto, Hiroyuki Ikemoto, Yumi Nakata, Noriyuki Miyoshi, Mamoru Isemura, Norio Ohashi, and Shinjiro Imai

Supplementary Figure 1

JNK Phospho JNK

1 2 3 4 5 6 2 3 4 5 6

54 kDa

46 kDa

Supplementary Figure 1: Full-length blots for Figure 3b. Western blot analysis of Rabbit IgG as positive control (lane 1), extracts from Jurkat cells, untreated control (lane 2), treated with PMA + A23187 (lane 3-6), treated with NIEGS (lane 4), LIEGS (lane 5) and LIEYS (lane 6), using SAPK/JNK antibody or Phospho-SAPK/JNK (Thr183/Tyr185) antibody.
